# Supplementary material for: Identification of Freezing-Responsive microRNAs and Their Targets in Chinese Jujube by Small RNA and Degradome Sequencing
Source: Int J Mol Sci. 2026 Sep 4;27(17):7912. doi: 10.3390/ijms27177912 (PMC13566890; doi:10.3390/ijms27177912)
Supplement: Supplementary file 1 [file ijms-27-07912-s001.zip › Supplementary Material S2/novel_miR_6.pdf]

| | | |
|---|---|---|
| Provisional ID | : | novel_miR_6 |
| Score total | : | 1459 |
| Score for star read(s) | : | 3.9 |
| Score for read counts | : | 1452.6 |
| Score for mfe | : | 0.9 |
| Score for randfold | : | 1.6 |
| Score for cons. seed | : | |
| Total read count | : | 2861 |
| Mature read count | : | 2054 |
| Loop read count | : | 5 |
| Star read count | : | 802 |

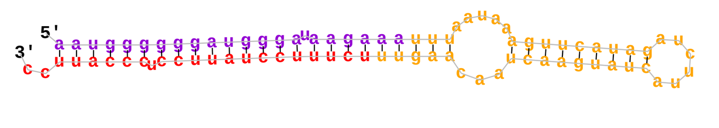

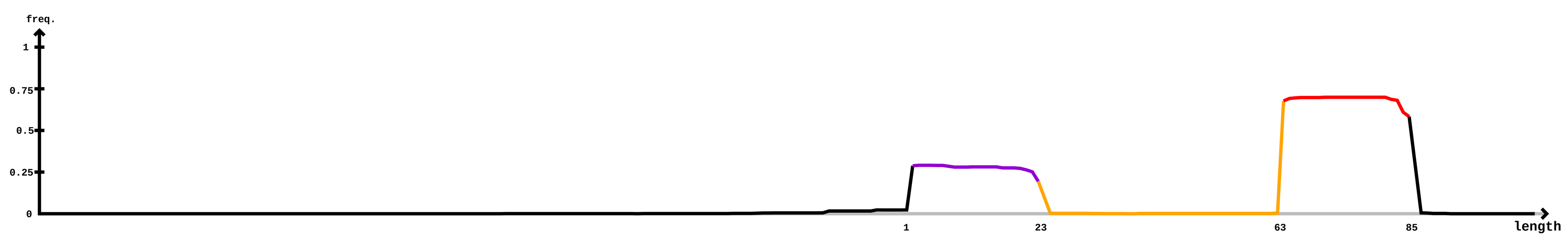


Star Mature

```
5'- agacaacuuuuuauauggucggucccaaaaucuuccucuugaaaacccuagcaaagcaaauuugcaaaaacgcauaucggcagagaaaaaaaaauugaaaacagagagacgggaaagguagcaaagaguuaggagguuauuaggaaugggggggaugggauaagaaauuuaauaaaguucauagaucuuacuaugaacuaacaaguuucuuuccuauuccucccauuccauucaauuuccuaauuuccuuc -3' obs
    agacaacuuuuuauauggucggucccaaaaucuuccucuugaaaacccuagcaaagcaaauuugcaaaaacgcauaucggcagagaaaaaaaaauugaaaacagagagacgggaaagguagcaaagaguuaggagguuauuaggaaugggggggaugggauaagaaauuuaauaaaguucauagaucuuacuaugaacuaacaaguuucuuuccuauuccucccauuccauucaauuuccuaauuuccuuc     exp
    .(((.(((........)))..)))........((((((((.........(((((.....)))))......((......))......................)))).))))((((......((((((((((((....((((((((((((((((.((((((((.....((((((((......)))))))))...)))))))))))))))))).))))))))....)))))))))))))))).
```

| read | reads | mm | sample |
|---|---|---|---|
| ucggcagagaaaaaaaaauuga | 2 | 0 | LCK-3 |
| aacagagagacgggaaagg | 3 | 0 | LCK-3 |
| aagguagcaaagaguuaggagguuauuagg | 1 | 0 | JT-3 |
| aagguagcaaagaguuaggagguuauuagg | 2 | 0 | JT-2 |
| uagcaaagaguuaggagguua | 1 | 0 | LT-1 |
| uagcaaagaguuaggagguuauuagg | 1 | 0 | JCK-3 |
| uagcaaagaguuaggagguuauuagg | 2 | 0 | JCK-2 |
| uagcaaagaguuaggagguuauuagg | 2 | 0 | LT-2 |
| agcaaagaguuaggagguuauuagg | 1 | 0 | LT-2 |
| agcaaagaguuaggagguuauuagg | 1 | 0 | JCK-2 |
| agcaaagaguuaggagguuauuagg | 2 | 0 | LT-3 |
| caaagaguuaggagguuauuaggaaug | 1 | 0 | LT-3 |
| uaggagguuauuaggaaug | 1 | 0 | JCK-1 |
| uaggagguuauuaggaaugggg | 1 | 0 | JCK-3 |
| aggagguuauuaggaauggg | 10 | 0 | JT-3 |
| aggagguuauuaggaauggg | 8 | 0 | JT-2 |
| aggagguuauuaggaaugggg | 8 | 0 | LCK-3 |
| aggagguuauuaggaaugggg | 4 | 0 | JCK-2 |
| aggagguuauuaggaaugggg | 1 | 0 | LT-2 |
| aggagguuauuaggaaugggC | 1 | 1 | JCK-2 |
| auuaggaaugggCggauggga | 16 | 1 | JT-1 |
| auuaggaaugggCggauggga | 1 | 1 | JCK-1 |
| auuaggaaugggggggaugggau | 1 | 0 | LT-1 |
| auuaggaaugggCggaugggauaagaa | 1 | 1 | JT-3 |
| gaaugggggggaugggauaaga | 1 | 0 | LT-3 |
| aaugggCggaugggauaa | 2 | 1 | JCK-1 |
| aaugggCggaugggauaa | 7 | 1 | JT-1 |
| aaugggCggaugggauaag | 3 | 1 | JT-3 |
| aaugggCggaugggauaag | 1 | 1 | JCK-3 |
| aaugggggggaugggauaag | 1 | 0 | JCK-2 |
| aaugggCggaugggauaag | 5 | 1 | JT-2 |
| aaugggggggaugggauaag | 6 | 0 | JT-2 |
| aaugggggggaugggauaag | 7 | 0 | JT-3 |

```
                                                                                                                                                Star                                          Mature
agacaacuuuuuauauggucggucccaaaaucuuccucuugaaaacccuagcaaagcaaauuugcaaaaacgcauaucggcagagaaaaaaaaauugaaaacagagagacgggaaagguagcaaagaguuaggagguuauuaggaauggggggaugggauaagaaauuuaauaaaguucauagaucuuacuaugaacuaacaaguuucuuuccuauuccucccauuccauucaauuuccuaauuuccuuc
................................................................................................................................................aauggggggaugggauaag.......................................................................................   3    0    LT-2
................................................................................................................................................aaugggCggaugggauaaga......................................................................................   8    1    JCK-2
................................................................................................................................................aauggggggaugggauaaga......................................................................................   3    0    LT-3
................................................................................................................................................aauggggggaugggauaaga......................................................................................   3    0    JCK-2
................................................................................................................................................aauggggggaugggauaaga......................................................................................   1    0    LT-1
................................................................................................................................................aaugggCggaugggauaaga......................................................................................   10   1    JCK-3
................................................................................................................................................aauggggggaugggauaaga......................................................................................   8    0    JT-1
................................................................................................................................................aauggggggaugggauaaga......................................................................................   1    0    JCK-1
................................................................................................................................................aaugggCggaugggauaagaa.....................................................................................   16   1    JT-3
................................................................................................................................................aauggggggaugggauaagaa.....................................................................................   14   0    JCK-2
................................................................................................................................................aauggggggaugggauaagaa.....................................................................................   8    0    JT-3
................................................................................................................................................aauggggggaugggauaagaa.....................................................................................   2    0    LT-1
................................................................................................................................................aauggggggaugggauaagaa.....................................................................................   7    0    JCK-3
................................................................................................................................................aaugggCggaugggauaagaa.....................................................................................   16   1    JCK-1
................................................................................................................................................aaugggCggaugggauaagaa.....................................................................................   25   1    JCK-3
................................................................................................................................................aauggggggaugggauaagaa.....................................................................................   13   0    LT-2
................................................................................................................................................aaugggCggaugggauaagaa.....................................................................................   13   1    JT-2
................................................................................................................................................aauggggggaugggauaagaa.....................................................................................   6    0    LCK-3
................................................................................................................................................aauggggggaugggauaagaa.....................................................................................   4    0    JT-2
................................................................................................................................................aaugggCggaugggauaagaa.....................................................................................   36   1    JCK-2
................................................................................................................................................aauggggggaugggauaagaa.....................................................................................   3    0    JCK-1
................................................................................................................................................aauggggggaugggauaagaa.....................................................................................   5    0    LT-3
................................................................................................................................................aaugggCggaugggauaagaaa....................................................................................   83   1    JCK-3
................................................................................................................................................aaugggCggaugggauaagaaa....................................................................................   35   1    JT-2
................................................................................................................................................aauggggggaugggauaagaaa....................................................................................   12   0    LT-1
................................................................................................................................................aauggggggaugggauaagaaa....................................................................................   14   0    LT-2
................................................................................................................................................aauggggggaugggauaagaaa....................................................................................   27   0    JCK-2
................................................................................................................................................aauggggggaugggauaagaaa....................................................................................   9    0    LT-3
................................................................................................................................................aauggggggaugggauaagaaU....................................................................................   1    1    JCK-3
................................................................................................................................................aauggggggaugggauaagaaU....................................................................................   3    1    LCK-3
................................................................................................................................................aauggggggaugggauaagaaU....................................................................................   2    1    JCK-2
................................................................................................................................................aaugggCggaugggauaagaaa....................................................................................   66   1    JCK-1
................................................................................................................................................aauggggggaugggauaagaaa....................................................................................   6    0    JT-3
................................................................................................................................................aauggggggaugggauaagaaa....................................................................................   14   0    JCK-3
................................................................................................................................................aauggggggaugggauaagaaa....................................................................................   10   0    JT-1
................................................................................................................................................aauggggggaugggauaagaaa....................................................................................   5    0    JT-2
................................................................................................................................................aaugggCggaugggauaagaaa....................................................................................   62   1    JT-3
................................................................................................................................................aauggggggaugggauaaAaaa....................................................................................   1    1    JCK-2
................................................................................................................................................aauggggggaugggauaagaaC....................................................................................   2    1    LT-3
................................................................................................................................................aaugggCggaugggauaagaaa....................................................................................   73   1    JT-1
................................................................................................................................................aaugggCggaugggauaagaaa....................................................................................   107  1    JCK-2
................................................................................................................................................aauggggggaugggauaagaaa....................................................................................   12   0    JCK-1
................................................................................................................................................aauggggggaugggauaagaaU....................................................................................   1    1    JCK-1
................................................................................................................................................aauggggggaugggauaagaaUu...................................................................................   1    1    LT-1
................................................................................................................................................aaugggCggaugggauaagaaau...................................................................................   3    1    JCK-1
................................................................................................................................................aaugggCggaugggauaagaaau...................................................................................   1    1    JCK-3
................................................................................................................................................aaugggCggaugggauaagaaau...................................................................................   3    1    JCK-2
................................................................................................................................................aaugggCggaugggauaagaaauu..................................................................................   1    1    JCK-1
................................................................................................................................................aaugggCggaugggauaagaaauu..................................................................................   2    1    JCK-3
.................................................................................................................................................augggggaugggauaagaaa....................................................................................   1    0    JCK-3
.................................................................................................................................................augggggaugggauaagaaa....................................................................................   2    0    JCK-1
.................................................................................................................................................augggCggaugggauaagaaa....................................................................................   5    1    JCK-2
......................................................................................................................................................Cggaugggauaagaaauuuaauaa............................................................................   1    1    JCK-2
......................................................................................................................................................Cggaugggauaagaaauuuaauaa............................................................................   2    1    JCK-3
..........................................................................................................................................................ugggauaagaaCuuuaauaaag..........................................................................   1    1    JCK-1
..........................................................................................................................................................ugggauaagaaauuuaauaaag..........................................................................   1    0    JCK-3
..........................................................................................................................................................ugggauaagaaauuuaauaaaguuca......................................................................   2    0    LCK-3
......................................................................................................................................................................................gaucuuacuaugaacuaacaaguA............................................   5    1    LCK-3
.............................................................................................................................................................................................................uucuuuccuauuccucccauuc.......................   1    0    JCK-3
.............................................................................................................................................................................................................uucuuuccuauuccucccauuc.......................   5    0    LT-2
.............................................................................................................................................................................................................Cucuuuccuauuccucccauucc......................   1    1    JCK-3
.............................................................................................................................................................................................................Cucuuuccuauuccucccauucc......................   1    1    JCK-2
..............................................................................................................................................................................................................ucuuuccuauuccuccca..........................   7    0    JT-3
..............................................................................................................................................................................................................ucuuuccuauuccuccca..........................   1    0    JCK-3
..............................................................................................................................................................................................................ucuuuccuauuccuccca..........................   5    0    LT-1
..............................................................................................................................................................................................................ucuuuccuauuccuccca..........................   3    0    JT-2
..............................................................................................................................................................................................................ucuuuccuauuccuccca..........................   15   0    LT-3
..............................................................................................................................................................................................................ucuuuccuauuccuccca..........................   3    0    LCK-3
..............................................................................................................................................................................................................ucuuuccuauuccucccU..........................   2    1    LT-3
..............................................................................................................................................................................................................ucuuuccuauuccuccca..........................   1    0    JCK-2
```

```
                                                                                                                                                         Star                                                      Mature
agacaacuuuuuauauggucggucccaaaaucuuccucuugaaaacccuagcaaagcaaauuugcaaaaacgcauaucggcagagaaaaaaaaauugaaaacagagagacgggaaagguagcaaagaguuaggagguuauuaggaauggggggaugggauaagaaauuuaauaaaguucauagaucuuacuaugaacuaacaaguuucuuuccuauuccucccauuccauucaauuuccuaauuuccuuc
...................................................................................................................................................................................................ucuuuccuauuccucccau.........................   3     0    LT-2
...................................................................................................................................................................................................ucuuuccuauuccucccau.........................   7     0    LCK-3
...................................................................................................................................................................................................ucuuuccuauuccucccUu.........................   1     1    LT-1
...................................................................................................................................................................................................ucuuuccuauuccucccau.........................   4     0    LT-3
...................................................................................................................................................................................................ucuuuccuauuccucccau.........................   2     0    LT-1
...................................................................................................................................................................................................ucuuuccuauuccucccauu........................   27    0    LT-3
...................................................................................................................................................................................................ucuuuccuauuccucccCuu........................   1     1    LT-2
...................................................................................................................................................................................................ucuNuccuauuccucccauu........................   1     1    JCK-1
...................................................................................................................................................................................................ucuuuccuauuccucccauu........................   29    0    JCK-2
...................................................................................................................................................................................................ucuuuccuauuccucccauu........................   16    0    JCK-1
...................................................................................................................................................................................................ucNuuccuauuccucccauu........................   1     1    LT-2
...................................................................................................................................................................................................ucuuuccuauuccucccauu........................   41    0    LCK-3
...................................................................................................................................................................................................ucuuuccuauuccucccauu........................   31    0    LT-2
...................................................................................................................................................................................................ucuuuccuauuccucccauG........................   1     1    JCK-1
...................................................................................................................................................................................................ucuuuccuCuuccucccauu........................   1     1    LT-3
...................................................................................................................................................................................................ucuuuccuauuccucccauu........................   20    0    LT-1
...................................................................................................................................................................................................ucuGuccuauuccucccauu........................   1     1    JCK-1
...................................................................................................................................................................................................ucuuuccuauuccucccauu........................   25    0    JCK-3
...................................................................................................................................................................................................ucuuuccuauuccucccauuc.......................   6     0    LT-1
...................................................................................................................................................................................................ucuuuccuauuccucccUuuc.......................   1     1    LT-1
...................................................................................................................................................................................................ucuuuccuauuccucccauuc.......................   1     0    JT-2
...................................................................................................................................................................................................ucuuuccuauuccucccauuc.......................   14    0    JCK-2
...................................................................................................................................................................................................ucuuuccuauuccucccCuuc.......................   1     1    JCK-2
...................................................................................................................................................................................................ucuuuccuauuccucccauuA.......................   1     1    JCK-1
...................................................................................................................................................................................................ucuuuccuauuccucccauuc.......................   12    0    LT-2
...................................................................................................................................................................................................ucuuuccuauuccucccauuc.......................   12    0    JCK-1
...................................................................................................................................................................................................ucuuuccuauuccucccauuc.......................   1     0    JT-3
...................................................................................................................................................................................................ucuuuccuauuccucccauuc.......................   8     0    LT-3
...................................................................................................................................................................................................ucuuuccuauuccucccauuc.......................   11    0    JCK-3
...................................................................................................................................................................................................ucuuuccuauuccucccauucc......................   87    0    LT-2
...................................................................................................................................................................................................ucuuuccuauuccucccauucc......................   250   0    JCK-1
...................................................................................................................................................................................................ucuuuccuauuccucccCuucc......................   1     1    LCK-3
...................................................................................................................................................................................................ucuuuccuauuccucccCuucc......................   1     1    JCK-1
...................................................................................................................................................................................................ucuCuccuauuccucccauucc......................   1     1    JCK-2
...................................................................................................................................................................................................ucuuuccuauuccucccauucc......................   140   0    LT-3
...................................................................................................................................................................................................ucuuuccuCuuccucccauucc......................   1     1    JCK-1
...................................................................................................................................................................................................ucuuuccuauuccucccauucc......................   64    0    LCK-3
...................................................................................................................................................................................................ucuuuccuauuccucccCuucc......................   2     1    JCK-2
...................................................................................................................................................................................................ucuuuccuauucAucccauucc......................   1     1    JCK-3
...................................................................................................................................................................................................ucuuuccuauuccucccauuUc......................   1     1    JCK-1
...................................................................................................................................................................................................ucuuuccuauuAcucccauucc......................   1     1    LT-3
...................................................................................................................................................................................................uUuuuccuauuccucccauucc......................   2     1    JCK-1
...................................................................................................................................................................................................ucuuuccuauuccucccauucc......................   3     0    JT-2
...................................................................................................................................................................................................uNuuuccuauuccucccauucc......................   1     1    JCK-2
...................................................................................................................................................................................................uUuuuccuauuccucccauucc......................   1     1    JCK-2
...................................................................................................................................................................................................ucuuuccuauuccucccauucU......................   1     1    JCK-3
...................................................................................................................................................................................................ucuuuccuauucAucccauucc......................   1     1    LT-3
...................................................................................................................................................................................................ucuuuccCauuccucccauucc......................   1     1    JT-1
...................................................................................................................................................................................................ucuuucUuauuccucccauucc......................   1     1    JCK-2
...................................................................................................................................................................................................ucuuuccuauuccucccauucc......................   13    0    JT-3
...................................................................................................................................................................................................ucuuuccuauuccucccauucc......................   527   0    JCK-2
...................................................................................................................................................................................................ucuuuccuauucUucccauucc......................   2     1    JCK-2
...................................................................................................................................................................................................ucuuuccuauuccucccauucc......................   22    0    JT-1
...................................................................................................................................................................................................ucuuuccuCuuccucccauucc......................   1     1    LT-1
...................................................................................................................................................................................................uUuuuccuauuccucccauucc......................   2     1    JCK-3
...................................................................................................................................................................................................ucuuuccuauuUcucccauucc......................   1     1    JCK-3
...................................................................................................................................................................................................ucuuuccuauuccucccauuNc......................   2     1    JCK-1
...................................................................................................................................................................................................ucuuuccuauuccucccCuucc......................   1     1    LT-2
...................................................................................................................................................................................................ucuuuccuauuccucccauucc......................   388   0    JCK-3
...................................................................................................................................................................................................ucuuuccuCuuccucccauucc......................   3     1    JCK-3
...................................................................................................................................................................................................ucuuuccCauuccucccauucc......................   1     1    JCK-2
...................................................................................................................................................................................................ucuuuccuauuccucccauucc......................   123   0    LT-1
...................................................................................................................................................................................................ucuuuccuauuccucccauucUa.....................   1     1    LT-3
...................................................................................................................................................................................................ucuuuccuauuccucccauucca.....................   2     0    LT-1
...................................................................................................................................................................................................ucuuuccuauuccucccCuucca.....................   1     1    JT-1
...................................................................................................................................................................................................ucuuuccuauuccucccauuccC.....................   4     1    LT-2
...................................................................................................................................................................................................ucuuuccuauuccucccauucca.....................   2     0    LT-3
...................................................................................................................................................................................................ucuuuccuauuccucccauucca.....................   11    0    JT-1
...................................................................................................................................................................................................ucuuuccuauuccucccauuccau....................   1     0    JCK-1
....................................................................................................................................................................................................cuuuccuauuccucccauu........................   1     0    JCK-3
```

```
                                                                                                                                                                          Star                                                                   Mature
agacaacuuuuuauauggucggucccaaaaucuuccucuugaaaacccuagcaaagcaaauuugcaaaaacgcauaucggcagagaaaaaaaaauugaaaacagagagacgggaaagguagcaaagaguuaggagguuauuaggaauggggggaugggauaagaaauuuaauaaaguucauagaucuuacuaugaacuaacaaguuucuuuccuauuccucccauuccauucaauuuccuaauuuccuuc
...............................................................................................................................................................................................................cuuuccuauuccucccauu........................   11   0   LCK-3
...............................................................................................................................................................................................................cuuuccuauuccucccauuc.......................   1    0   LT-3
...............................................................................................................................................................................................................cuuuccuauuccucccauuU.......................   3    1   LT-2
...............................................................................................................................................................................................................cuuuccuauuccucccauucc......................   2    0   LT-1
...............................................................................................................................................................................................................cuuuccuauuccucccauucc......................   4    0   JCK-1
...............................................................................................................................................................................................................cuuuccuauuccucccauucc......................   3    0   JCK-3
...............................................................................................................................................................................................................cuuuccuauuccucccauucc......................   10   0   JCK-2
...............................................................................................................................................................................................................cuuuccuauuccucccauucc......................   5    0   LT-3
...............................................................................................................................................................................................................cuuuccuauuccucccauuccauu...................   4    0   LT-2
................................................................................................................................................................................................................uuuccuauuccucccauu........................   2    0   JCK-3
................................................................................................................................................................................................................uuuccuauuccucccauu........................   1    0   JCK-1
................................................................................................................................................................................................................uuuccuauuccucccauucc......................   1    0   JCK-3
................................................................................................................................................................................................................uuuccuauuccucccauucc......................   1    0   LT-3
................................................................................................................................................................................................................uuuccuCuuccucccauucc......................   1    1   LT-3
................................................................................................................................................................................................................uuuccuauuccucccauucc......................   1    0   LT-1
................................................................................................................................................................................................................uuuccuauuccucccauucc......................   3    0   JCK-1
.................................................................................................................................................................................................................uuccuauuccucccauucc......................   1    0   LT-3
.................................................................................................................................................................................................................uuccuauuccucccauuccaC....................   2    1   JCK-2
.................................................................................................................................................................................................................uuccuauuccucccauuccauu...................   1    0   JCK-3
.................................................................................................................................................................................................................uuccuauuccucccauuccauu...................   2    0   JCK-1
.....................................................................................................................................................................................................................uauuccucccauuccauucaa................   2    0   JCK-2
.....................................................................................................................................................................................................................uauuccucccauuccauucaa................   1    0   JCK-3
.....................................................................................................................................................................................................................uauuccucccauuccauucaa................   2    0   JCK-1
```
